# From etiology to precision oncology: a systematic review of the causes and evolving treatments of prostate cancer

**DOI:** 10.3389/fonc.2026.1941788

**Published:** 2026-10-07

**Authors:** Nakitto Irene Kisakye, Geoffrey Ayebazibwe, Paula Mary Amito, Patricia D. Ajambo, Michael Tirwomwe, Annet Tiko, Tusingwire Pliers, Asaph Owamukamaeli, Timothy Nduhukire, Ngabirano Chrispuss, Immaculate Mandera, Racheal Owomugisha, Swase Dominic Terkimbi

**Affiliations:** 1 Kabale University School of Medicine, Kabale, Uganda; 2 Victoria University, Kampala, Uganda

**Keywords:** androgen deprivation therapy, biomarkers, etiology, immunotherapy, precision oncology, prostate cancer, risk factors, systematic review

## Abstract

**Background:**

Prostate cancer remains one of the most frequently diagnosed malignancies and a leading cause of cancer-related mortality among men globally. Its development is driven by a complex interaction of genetic susceptibility, molecular alterations, hormonal signaling, environmental exposures, and lifestyle-related factors. This systematic review synthesizes current evidence on the etiological mechanisms and evolving therapeutic landscape of prostate cancer.

**Methods:**

A systematic review was conducted according to the Preferred Reporting Items for Systematic Reviews and Meta-Analyses (PRISMA) 2020 guidelines. Literature published between January 2010 and December 2025 was searched in PubMed, Scopus, and Web of Science databases. Eligible studies included original research, clinical studies, translational studies, and experimental investigations evaluating prostate cancer etiology, molecular mechanisms, risk factors, biomarkers, or therapeutic interventions.

**Results:**

The review included 29 studies encompassing epidemiological cohorts, clinical studies, genomic analyses, tissue-based investigations, and preclinical experimental studies. Evidence demonstrated that prostate cancer etiology involves multiple interconnected pathways, including androgen receptor signaling, DNA repair alterations, genomic instability, chromatin remodeling, metabolic reprogramming, and inflammatory mechanisms. Genetic variants, including single nucleotide polymorphisms and polygenic risk scores, were associated with disease susceptibility, aggressiveness, treatment response, and toxicity outcomes. Therapeutic advances included optimization of androgen deprivation therapy, novel androgen receptor pathway inhibitors, chemotherapy, radiotherapy personalization, bone-targeted therapies, PSMA-directed radioligand therapy, and emerging immunotherapeutic approaches. Biomarker-driven strategies, including genomic classifiers and molecular signatures, showed potential for improving risk stratification and individualized treatment selection.

**Conclusion:**

Prostate cancer is a biologically heterogeneous disease requiring integrated approaches that combine molecular understanding with clinical decision-making. Recent advances in precision oncology have expanded therapeutic options beyond conventional treatment paradigms, enabling more personalized management strategies. However, further research is required to improve representation of diverse populations, validate biomarkers across different ethnic groups, and optimize the clinical implementation of emerging targeted and immune-based therapies.

**Systematic Review Registration:**

https://www.crd.york.ac.uk/PROSPERO/, identifier CRD420261435105.

## Introduction

### Background

Prostate cancer remains one of the most common malignancies and a major cause of cancer-related deaths in men worldwide (1). Its etiology is multifactorial, encompassing genetic, hormonal, environmental, and lifestyle determinants, while treatment modalities continue to evolve toward precision-based and immunogenomic strategies (2). Prostate cancer remains a major global health challenge, standing as one of the most frequently diagnosed malignancies and a leading cause of mortality among men worldwide (1). Its impact increased by a rising incidence in both developed and developing nations, compounded with the complexities of managing a disease that can range from indolent to highly aggressive and treatment-resistant (3). The cause of prostate cancer is not attributed to a single cause but rather arises from a complex interplay of associated risk factors. These include inherited genetic predispositions, hormonal imbalances, chronic inflammation, and modifiable lifestyle and environmental exposures (4). Understanding this multifactorial nature is crucial for developing effective prevention and early intervention strategies (4).

Also, the therapeutic options for prostate cancer have undergone a profound change. Treatment has transitioned from a reliance on localized interventions like radical prostatectomy and radiotherapy for early stage disease to a multimodal approach for advanced and metastatic cases (5). While androgen deprivation therapy (ADT) has long been the foundation of systemic treatment, its efficacy is often limited by the development of castration resistant disease (6). In response, the field has witnessed remarkable progress, with the advent of novel hormonal agents, potent chemotherapeutics, and, more recently, precision medicine strategies (7). Immunotherapies and targeted agents, including PARP inhibitors for tumors with DNA repair defects and PSMA-targeted radioligand therapies, are now offering immerging avenues of hope, particularly for patients with advanced or previously refractory disease (8).

Given the rapid pace of discovery and the expanding ability of therapeutic options, a comprehensive synthesis of current evidence is essential (9). This systematic review is focused on consolidated and critically evaluated the peer reviewed literature published between 2010 and 2025 on the major causes and treatment options of prostate cancer. By integrating genetic, molecular, and clinical findings, we seek to provide a clear overview of the disease’s causes and the evolving standards of care, highlighting the promising translational links between mechanistic understanding and clinical application.

### Research question

#### Primary question

What is the current evidence on the major causes and treatment modalities of prostate cancer, integrating genetic, molecular, and clinical findings?

#### Objective

To systematically review current evidence on the major causes and treatment modalities of prostate cancer, integrating genetic, molecular, and clinical findings published between 2010 and 2025.

## Methodology

This systematic review was registered on PROSPERO and the link to the published protocol is this https://www.crd.york.ac.uk/PROSPERO/view/CRD420261435105. This review followed the PRISMA 2020 guidelines (**Figure 1**). Searches were performed in PubMed, Scopus, and Web of Science for peer-reviewed studies from January 2010 to December 2025 using keywords: “prostate cancer,” “etiology,” “risk factors,” “treatment,” “therapy,” and “management.” Eligible studies included original research, clinical trials, and meta-analyses focusing on etiological mechanisms or therapeutic interventions for prostate cancer. Data was extracted and narratively synthesized according to two domains: *causation* and *treatment*.

**Figure 1:**
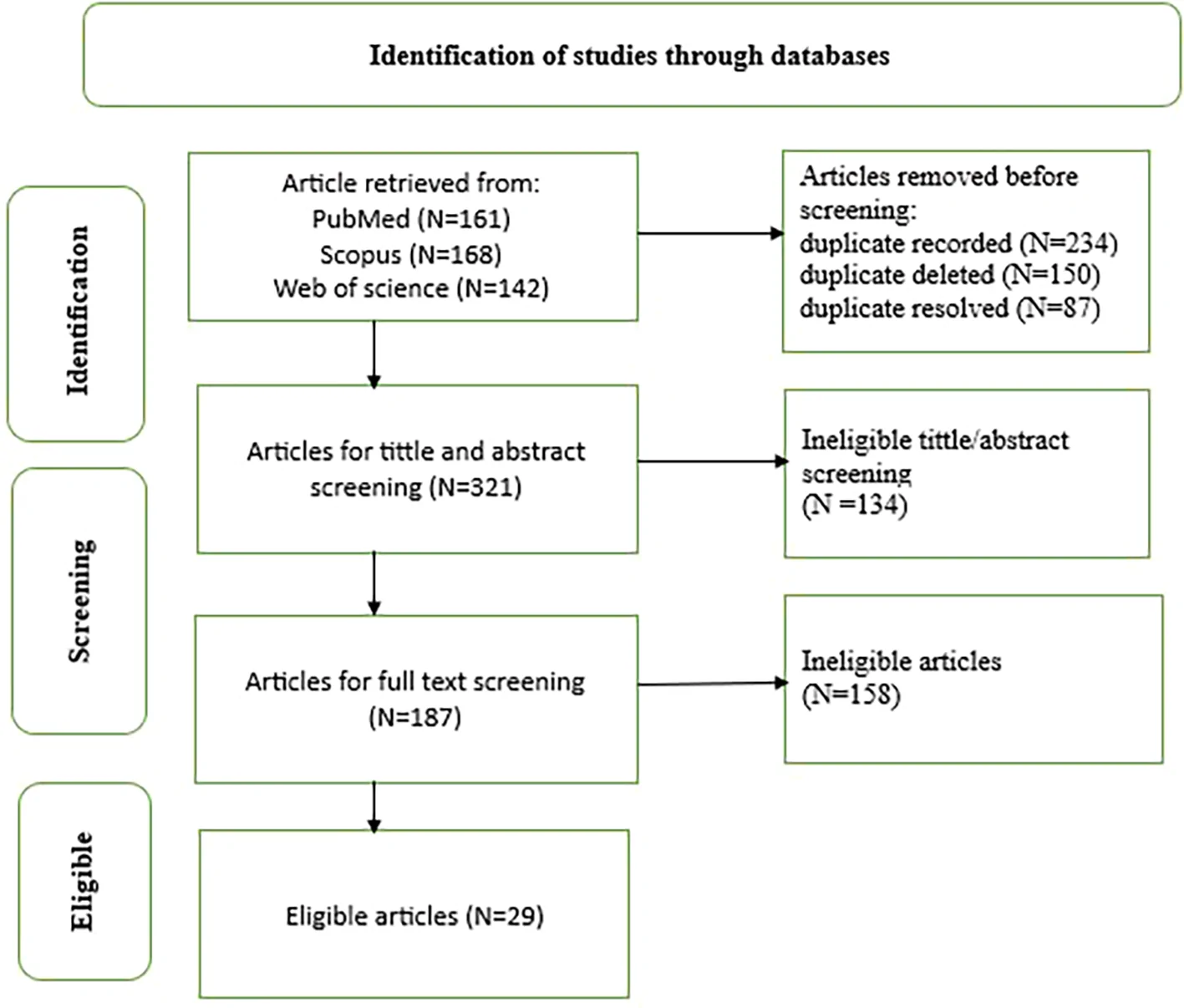
The PRISMA flow diagrams.

### Data sources and search strategy

Research articles from January 2010 to December 2025 from PubMed, Scopus, and Web of Science databases were searched for literature. The search terms combined keywords for prostate cancer, risk factors, etiology, and treatment using Boolean operators and MeSH terms.

Search terms: (“prostate cancer” OR “prostatic carcinoma”) AND (“etiology” OR “risk factors” OR “causes”) OR (“treatment” OR “therapy” OR “management”). Time frame: 2010–2025 and no language filters were used during the search. The retrieved papers were exported to excel sheet for further management. Due to the abundance of the publications on the subject matter, grey literature and manual search was not done.

Eligibility Criteria:

Population: Prostate cancer.

Interventions: Treatment modalities for prostate cancer.

Comparators: placebo, surgeries, or therapies.

Outcomes: Improved prostate cancer management.

Study types: *in vivo* controlled studies.

### Defined scope

#### Inclusion criteria

- Original peer-reviewed studies and meta-analyses.
- Research addressing causes, risk factors, or treatments of prostate cancer.

#### Exclusion criteria

- Reviews, commentaries, and conference abstracts.
- Studies without primary data or irrelevant to prostate cancer.

### Data extraction and analysis

Data was extracted by two reviewers independently on study design, sample properties, compound, dose, duration, outcomes, and findings. Consensus was used to resolve discrepancies. Two reviewers were involved independently and any disagreements were resolved using a third reviewer. The interviewer agreement was taken at 0.80 Cohen’s Kapa. Discrepancy was resolved and the third person had a final verdict where consensus was not reached. Variables included study design, mean age, population, etiological focus, treatment type, and clinical outcomes.

### Data synthesis

The study properties, were grouped into etiological and therapeutic domains for descriptive synthesis. Meta-analysis was not performed due to heterogeneity in the treatment modalities for prostate cancer.

### Quality assessment

For Human Studies: We applied the Cochrane RoB 2 tool for randomized trials. The risk of bias assessment was conducted by the two reviewers who performed the screening. This process involved the following steps:

1. Each reviewer independently assessed the risk of bias for all included studies using the appropriate tools.
2. For each domain, a judgment of “Low risk,” “High risk,” or “Unclear risk” was be assigned. Low risk: The study appeared to have adequately addressed the potential for bias in this area. High risk: There was a significant risk of bias that could substantially affect the results. Unclear risk: The study reported lacks sufficient information to make a clear judgment.

For Randomized Controlled Trials (RCTs): Cochrane RoB 2 tool.

1. Select the Result for Assessment: Risk of bias was assessed for a specific result from a study, not for the study as a whole. Focus on the results that were used for the review’s primary conclusions, typically those in the ‘Summary of Findings’ table (e.g., effect on sperm count, testosterone levels, or glycemic control).
2. Define the Effect of Interest: We determined whether the review aims to assess the effect of assignment to intervention (the ‘intention-to-treat’ effect) or the effect of adhering to the intervention as specified in the protocol (the ‘per-protocol’ effect).
3. Independent Assessment by Two Reviewers: Two reviewers independently assessed the risk of bias for each selected result. This minimized reviewer bias and ensured the reliability of the assessment.
4. Answer Signaling Questions and Assign Domain Judgements: Reviewers used the detailed signaling questions for each domain within the chosen tool to guide their assessment. These questions sought factual information from the study report. Response Options: Each signaling question was answered with one of the following: Yes/Probably Yes/Probably No/No/No Information. The responses to the signaling questions are mapped by an algorithm to a proposed domain-level judgement. Domain Judgements: Each domain was rated as one of the following: Low risk of bias; Some concerns (for RoB 2) or Moderate risk (for ROBINS-I) and High risk of bias (for RoB 2) or Serious/Critical risk (for ROBINS-I).
5. To determine the overall Risk of Bias Judgement: The overall risk of bias for a specific result was based on the least favorable assessment across the domains. RoB 2 Overall Judgement: Low risk of bias: All domains are at low risk. Some concerns: At least one domain has ‘Some concerns’, but no domain is at high risk. High risk of bias: At least one domain is at high risk, *or* there are ‘Some concerns’ for multiple domains that substantially lowers confidence in the result.

## Results

### Study selection and characteristics

A systematic review search identified 29 potentially relevant articles on prostate cancer etiology, risk factors, treatment, therapy, and management from 2010 to 2026 (**Table 1**). After full text review, 29 articles met the inclusion criteria. The included studies comprised diverse designs including retrospective cohort studies (n=13), prospective cohort studies (n=4), preclinical *in vitro*/*in vivo* studies (n=8), tissue microarray studies (n=2), and registry-based studies (n=1).

**Table 1:** Study.

No	Author	Year	Country	Study design	Sample size	Duration of the study	Intervention	Comparator	Key primary outcomes	Secondary outcomes
1	(10)	2025	Multi-country	Multi-cohort GWAS with Prospective Cohort Validation	Binarized PRS (upper decile vs rest): PRShigh defined as top 10%	Same as above	Radiotherapy for non-metastatic prostate cancer	PRSlow (remaining 90%)	Time-to-patient-reported gross hematuria (≥ Grade 2)	Urinary retention (≥G2), urinary frequency (≥G2); clinically-diagnosed irradiation cystitis (UKB); gene-burden testing
2	(11)	2020	China	*In vitro* Laboratory Study (Cell-based Experimental Study)	PCa cell lines: PC3, DU145, LNCaP; Normal: HPE cells	Not applicable (*in vitro* study)	DTX3L overexpression (pcDNA-DTX3L transfection)	Vector control (pcDNA 4.1); shRNA negative control	Radioresistance (cell survival after ionizing radiation 0–8 Gy)	DNA damage repair markers (γH2AX, Rad51); AKT pathway activation (p-Akt/Akt); Effect of AKT inhibition (MK-2206)
3	(12)	2017	Spain	Retrospective Case-Control Study with Prospective Follow-up	Total: 311 (155 controls, 156 patients)	Median follow-up: 34.18 months (range: 1–54 months)	N/A (Observational genetic association study)	N/A (Case-control design)	Association between 9 SNPs in sex hormone genes and PCa aggressiveness	Biochemical recurrence-free survival (BRFS) by treatment type; Multigene analysis for risk stratification
4	(13)	2020	USA	Retrospective Cohort (Targeted Sequencing Study)	Total: 205 AA men (all with RP)	Median follow-up: 5 years (IQR 2–10 years)	N/A (Observational genomic study)	N/A (No comparator group)	Association of somatic genomic alterations with metastasis in AA men	Association with biochemical recurrence (BCR); Percent Genome Alteration (PGA); p27 protein expression by IHC
5	(14)	2022	Germany	*In vitro* and *In vivo* Experimental Study	Multiple cell lines: PCa (DU145, PC3), HNSCC (FaDu, Cal33, SAS, UT-SSC-14), GBM (LN229, U87MG); Primary cultures (n=4-6)	Up to 21 days post-irradiation	Proton beam irradiation (single dose: 4–8 Gy; fractionated: 24 Gy in 5 fractions)	Photon irradiation (single dose: 4–8 Gy; fractionated: 26 Gy in 5 fractions)	CSC marker dynamics (ALDH, CD44, CD133, CXCR4); Clonogenic survival; Sphere-forming capacity; DNA damage (ɣH2AX foci); Cellular plasticity rates (mathematical modeling)	Senescence (β-galactosidase); Apoptosis (Annexin V, caspase-3/7); Autophagy; Histone methylation (H3K4me3, H3K36me3, H3K27me3); Transcriptome changes; DNA methylation; TCA metabolome
6	(15)	2020	Brazil	Retrospective Cohort (Biomarker Study)	Total: 60 PCa patients (all treated with RP)	Sample collection 2014-2015	N/A (Observational biomarker study)	N/A (No comparator group)	Association of 8 cfmRNA markers with high-risk PCa (Gleason ≥8 and/or pT3+)	Association with histopathological features (extracapsular extension, seminal vesicle invasion, tumor stage)
7	(16)	2020	USA	Cross-Sectional Genomic Study (Retrospective)	Total: 327 (232 EAM, 95 AAM)	Not applicable (cross-sectional)	N/A (Observational genomic comparison study)	N/A (Race-based comparison)	Differential gene expression by race for 3 commercial prognostic panels (Oncotype DX, Prolaris, Decipher); Estimated genomic risk scores by race	Intra-panel co-expression patterns by race; Correlation of risk scores with CAPRA-S and NCCN risk groups
8	(17)	2020	China	*In vitro* Laboratory Study + Bioinformatics Analysis	Cell lines: LNCaP, LNCaP-AI (androgen-independent), WPMY-1	Cell culture: 12+ months for LNCaP-AI establishment	AR signaling modulation (DHT stimulation, CDX/AR antagonist treatment); Wnt pathway inhibition (KY02111, XAV-939)	Androgen-dependent LNCaP cells	AR transcriptional activity in AIPC vs ADPC; Wnt pathway activation after ADT; LEF1 and WNT5A as direct AR targets; Wnt pathway role in AIPC growth	Effect of Wnt inhibitors on proliferation, cell cycle, apoptosis; Gene expression in PCa progression (GSE6919, TCGA)
9	(18)	2020	USA, Canada	Retrospective Cohort (Biomarker Development and Validation)	Total: 15,889 Training: 6,766 Test: 3,363 Validation 1 (RP): 5,062 Validation 2 (Bx): 698	Not applicable (cross-sectional)	N/A (Observational genomic study)	N/A (No comparator group)	Development of genomic SVI (gSVI) signature to predict seminal vesicle invasion at radical prostatectomy	Comparison with Partin Tables and MSKCC nomograms; AUC comparison; Decision curve analysis
10	(19)	2023	Australia, Ireland	*In vitro* Laboratory Study + Human Tissue Analysis	Tissue: >100 PCa patients (TMA) Cell lines: PNT2, LNCaP, PC3	Not applicable (cross-sectional tissue + *in vitro*)	Androgen treatment (R1881); siRNA knockdown (sortilin, AS160); Glucose modulation; Lipid treatment	Vehicle control; Scrambled siRNA; Normal glucose (5.5 mM)	Sortilin and syndecan-1 expression patterns in PCa tissue; Role of sortilin in glucose metabolism; Role of syndecan-1 in lipid metabolism; Androgen regulation of metabolic switch	GLUT1/GLUT4 trafficking; AS160/Rab10/Rab14 pathway; Progranulin and LPL interactions; β3 integrin colocalization; Syndecan-1 cleavage products
11	(20)	2022	Germany, Poland	Retrospective Cohort + *In vitro* + Liquid Biopsy	Tissue: 199 primary tumors CTC: 38 blood samples (32 patients) TCGA: 488 patients	Not applicable	RAI2 knockdown (shRNA); AR knockdown; AR inhibition (bicalutamide, enzalutamide, hormone deprivation)	Non-target shRNA; vehicle control	RAI2 expression in PCa tissue and CTCs; Correlation with AR pathway; Corepressor function of RAI2; Association with biochemical recurrence	Correlation with hypoxia scores, genomic instability, epithelial markers; CTC gene expression profiling; RAI2-AR interdependence
12	(21)	2023	France, Spain	Retrospective Cohort + *In vitro* + *In vivo*	Tissue: 52 primary PCa + 4 NEPC Cell lines: VCaP, LNCaP, PC3, PC3M, DU145, NCI-H660	Not applicable	FSCN1 knockdown (siRNA/shRNA); AR overexpression; DHT treatment; Bicalutamide treatment	Control siRNA/shRNA; Vehicle control	FSCN1 expression in PCa and NEPC; AR-mediated FSCN1 repression; Role of FSCN1 in NE differentiation; FSCN1 knockdown effects on tumor growth	FSCN1 correlation with KLK3; Neuroendocrine marker expression; Invadopodia formation; AR and FOXA1 binding to FSCN1 regulatory regions
13	(22)	2021	USA, Switzerland, China	*In vitro* + *In vivo* Laboratory Study	Cell lines: LAPC4, VCaP, LNCaP, LAPC4-CR, VCaP-CR, LNCaP-abl	Not applicable (cell line establishment)	Castration-resistant cell line development; AR antagonist treatment (enzalutamide, bicalutamide); DHT treatment	Parental androgen-dependent lines (LAPC4, VCaP, LNCaP)	Castration-resistant growth *in vitro* and *in vivo*; Response to AR antagonists; Response to high-dose androgen; Transcriptomic and proteomic profiling	AR and NKX3.1 expression; MAPK and AKT phosphorylation; Neuroendocrine markers; Differential gene/protein expression
14	(23)	2017	South Korea	Prospective Cohort (Genetic Association Study)	Total: 912 PCa patients (all treated with RP)	Median follow-up: 51 months	N/A (Observational genetic study)	N/A (No comparator group)	Association of GRS with BCR after RP; Development of clinico-genetic prediction model	Identification of 16 SNPs associated with BCR; Comparison of AUC with/without GRS; Subgroup analysis in pT3 and pT2R1 patients
15	(24)	2019	China	*In vitro* + *In vivo* Laboratory Study + Human Tissue Analysis	Tissue: 64 PCa patients + 14 BPH controls Cell lines: PC-3, DU145, 22Rv1, LNCaP, RWPE-1 Animal: Nude mice	Not applicable	HMGB1 knockdown (shRNA/siRNA); HMGB1 overexpression (lentiviral); BRG1 modulation	sh-Ctrl; vector control	HMGB1 role in PCa proliferation, migration, invasion; HMGB1-BRG1 interaction; Akt pathway activation; EMT regulation; *In vivo* tumor growth and metastasis	Clinical correlation of HMGB1/BRG1 with PCa prognosis; Cell cycle analysis; Protein-protein interaction
16	(25)	2013	Portugal	Retrospective Case-Control + Prospective Follow-up Cohort	Total: 1,765 Cases: 891 PCa Controls: 874 Follow-up: 428 ADT patients	Median follow-up: 51 months	TGFBR2-875G>A genotyping; ADT treatment	N/A (Genetic association study)	TGFBR2-875G>A association with PC risk and CRPC development; TGFBR2 mRNA expression by genotype; Predictive model improvement	Association with Gleason score; CR-free survival; C-index analysis
17	(26)	2018	China	Retrospective Cohort (Tissue Microarray Study)	Total: 456 PCa: 386 BPH: 60 Normal: 10 Lymph node: 30	January 2008 - January 2013 (5 years)	N/A (Observational biomarker study)	BPH and normal prostate tissues	Paxillin expression in PCa vs normal/BPH; Association with clinicopathological features; Association with lymph node metastasis; Association with biochemical recurrence	Predictive value for lymph node metastasis (ROC); Independent risk factors for LNM and BCR; Kaplan-Meier survival analysis
18	(J. 27)	2020	China	Retrospective Cohort (Tissue Microarray Study)	Total: 329 PCa: 279 BPH: 50 Lymph node: 40	January 2012 - September 2015; Median follow-up: 55 months (range 9-95)	N/A (Observational biomarker study)	BPH tissues	LIMK1 expression in PCa vs BPH; Association with clinicopathological features; Association with lymph node metastasis; Association with biochemical recurrence	LIMK1 in paired lymph node vs primary tumor; Independent risk factors for LNM and BCR; Kaplan-Meier survival analysis
19	(28)	2024	USA	*In vitro* + *In vivo* + PDX Analysis	Cell lines: C4-2B, C4-2BER, C4-2BAR, C4-2BDR, PC3, PC3-CR, 22RV1, LNCaP PDX: 8 specimens from 5 patients *In vivo*: NOD-SCID mice	Not applicable	Cabazitaxel; Ispinesib (kinesin inhibitor); CW069 (HSET inhibitor); SLCO1B3 knockdown	Vehicle control; Untreated cells	Cross-resistance mechanisms between anti-androgens and taxanes; Role of HSET in resistance; SLCO1B3 transporter function; Kinesin targeting to overcome resistance	Phenotypic reprogramming (EMT/MET); Gene expression profiling; PDX characterization; HSET-AR interaction
20	(29)	2018	Canada	*In vitro* + *In vivo* + Bioinformatics	Cell lines: PC3, DU145, PC3-IRR TCGA: 487 prostate tumors, 52 normal *In vivo*: NOD-SCID mice	Not applicable	miR-106a overexpression; LITAF siRNA knockdown; KU-55933 (ATM inhibitor)	Control mimic; Control siRNA; DMSO vehicle	miR-106a role in radioresistance; LITAF as miR-106a target; ATM regulation; KU-55933 radiosensitization	TCGA analysis; Proliferation; Senescence; Cell cycle; *In vivo* tumor growth; BCR-free survival
21	(30)	2013	Taiwan	Retrospective Cohort (Genetic Association Study)	Total: 645 advanced PCa patients treated with ADT	Mean follow-up: 39 months (range: 3-125)	ADT (orchiectomy or LHRH agonist ± antiandrogen)	N/A (Observational genetic study)	Association of 18 SNPs in sex hormone pathway genes with PCSM, ACM, and time to progression after ADT	Combined genotype effect on PCSM; Survival tree analysis for SNP-SNP interactions
22	(31)	2014	Canada	Retrospective Cohort (Biomarker Study)	Total: 293 IGRT: 139 RadP: 154	IGRT: median 7.9 years; RadP: median 4.8 years	Image-guided radiotherapy (IGRT) (dose 60-79.8 Gy)	Radical prostatectomy (RadP)	NBN copy number gains as predictor of bRFR after IGRT; Comparison with RadP cohort; Association with genomic instability (PGA)	DDR gene CNAs (MRE11A, RAD50, NBN, ATM, ATR, PRKDC); *In vitro* NBN expression and radioresistance; c-statistic improvement
23	(32)	2014	UK	Prospective Cohort (RNA-seq Study)	Total: 7 patients (paired pre/post ADT)	Mean time to second biopsy: 202 ± 143 days	ADT (androgen-deprivation therapy)	Pre-ADT tumor biopsies (paired)	ADT-regulated gene expression changes; Wnt/β-catenin pathway activation; XAV939 effects on CRPC cell growth	TMPRSS2-ERG fusion expression; b-catenin overexpression in CRPC (IHC); Pathway enrichment analysis
24	(33)	2022	South Korea	Retrospective Cohort (Genetic Association Study)	Total: 11,320 Training: 10,187 (2,702 cases, 7,485 controls) Validation: 1,133 (311 cases, 822 controls)	Not applicable	N/A (Observational genetic study)	N/A (No comparator group)	Development and validation of PRS for csPCa (GS ≥7) prediction in Koreans	Identification of 83 PCa-associated SNPs; Predictive performance by SNP number; Risk stratification by PRS percentile
25	(34)	2023	USA	Retrospective Cohort (Registry Study)	Total: 38,921 (post-QC) GG1: 14,331 GG2: 16,159 GG3: 5,661 GG4: 1,775 GG5: 995	2016-2021	N/A (Observational genomic study)	N/A (No comparator group)	Relationship between tumor volume (PPC) and genomic risk (Decipher GC) by GG	Correlation between PPC and GC across GG strata; Multivariable analysis of PPC predictors
26	(35)	2014	USA	Retrospective Cohort (Genetic Association Study)	Total: 1,321 white men (all treated with RP)	2003-2011	N/A (Observational genetic study)	N/A (No comparator group)	Association of 38 PC risk SNPs with tumor volume and prostate size at RP	Association of 8q24 SNPs with tumor volume; PSA-SNPs with tumor/prostate volume; LUTS-SNPs with volume
27	(36)	2022	Taiwan	Retrospective Cohort + *In vitro* + Bioinformatics	Total: 458 PCa patients (treated with RP)	Median follow-up: 54 months	N/A (Observational genetic study; TNFRSF13B knockdown *in vitro*)	N/A (No comparator group)	Association of 19 immunodeficiency pathway SNPs with BCR after RP; TNFRSF13B functional role	TNFRSF13B expression and prognosis; Cell proliferation/colony formation; Microarray gene expression profiling
28	(37)	2017	Taiwan	*In vitro* + Bioinformatics	TCGA: 498 PRAD + 52 normal Cell lines: PPC1 (PC-3), LNCaP, AU565, MCF7, MDA-MB361, MDA-MB231, ARPE19 Tissue: 4 PCa pts	Not applicable	TPX2 siRNA knockdown	si-CTL (scrambled siRNA)	TPX2 expression in PCa; Effect of TPX2 silencing on cell growth, colony formation, spheroid formation, cell cycle, apoptosis, micronucleation	TCGA expression analysis; Cell cycle PCR arrays; Protein expression changes (CDK1, cyclin B, securin, separase, aurora A)
29	(38)	2017	USA	Pilot Randomized Controlled Trial (2×2 factorial design)	32 participants (estimated; 8 per group)	12 weeks	RTPS: Periodized resistance training (3x/week, 45 min sessions) + 50g/day whey protein supplementation	PS: Protein supplementation only (50g/day whey protein)	Sarcopenic obesity (appendicular skeletal mass and body fat % via DXA)	Metabolic syndrome components (waist circumference, triglycerides, HDL-C, blood pressure, glucose), cardiometabolic biomarkers (insulin, IGF-1, HbA1c, CRP), physical function (muscular strength, 400m walk, timed up and go, stair climb, balance)

Study sample sizes ranged from small mechanistic studies (7–8 patients) to large epidemiological cohorts: Large cohorts (>500 patients): (25) included 891 prostate cancer patients and 874 healthy controls; (30) included 645 patients; (10) included 3,988 training, 2,034 test, and 8,430 validation cohort patients. Medium cohorts (100–500 patients): (26) included 386 patients; (J. 27) included 279 patients; (36) included 458 patients; (20) analyzed TCGA data from 488 patients. Small cohorts (<100 patients): (32) included 8 patients (7 for RNA-seq analysis); (24) analyzed 64 prostate cancer tissues.

The majority of studies were conducted in predominantly Asian (Chinese, Japanese, Taiwanese) or European/Caucasian populations, with limited diversity representation. The multinational study by (10) included predominantly European ancestry populations, highlighting a significant gap in understanding prostate cancer genetics across diverse ethnic groups. Studies predominantly included older adult male populations, consistent with the typical age distribution of prostate cancer (median ages ranging from 60–75 years across studies). Follow-up durations varied from short-term laboratory experiments to median follow-ups of 51 months (25) and 55 months (M. 39).

### Geographical relevance of included studies

The included studies encompassed diverse patient populations with varying demographic, clinical, and pathological characteristics. The studies were conducted across multiple geographic regions One study was multinational and the others were (8) United States, (6) in China, (3) in Taiwan, (3) in Canada, then remain countries each had one study (1) in Spain, Germany, Brazil, Australia, Ireland, Poland, South Korea, Portugal. These studies peaked in 2020 with (7), followed by (4) in both 2014 and 2022 each, then (3) in both year 2014 and 2023 each, then followed by (2) in both 2013 and 2018 each, then the remain 4 years had (1) study each for 2019, 2021, 2024 and 2025.

### Results on etiology and risk factors

Multiple studies recognized genetic variants contributing to prostate cancer etiology. (12) analyzed 9 SNPs in androgen and estrogen pathway genes (AR, CYP17A1, LHCGR, ESR1, ESR2) in 311 Caucasian men. While no SNPs were connected with prostate cancer risk, five variants were significantly associated with aggressive disease characteristics. (13) did targeted sequencing of 205 prostate tumors from African American men, revealing that the most common somatic mutations were SPOP (11.2%), FOXA1 (8.3%), and TP53 (3.9%). This was the first study correlating somatic genomic alterations with metastasis in African American men, addressing an important gap in understanding prostate cancer etiology in high-risk populations. (33) developed an ethnic-specific polygenic risk score (PRS) for clinically significant prostate cancer in Korean males. From 83 previously reported PCa-associated SNPs, rs72725879 in PRNCR1 showed the highest significance for PCa risk (OR = 0.597, p=4.3×10^-45^). (16) examined race-specific expression patterns of three commercial prognostic panels in 327 prostate cancer patients. Of 60 genes measured, 48% were differentially expressed by race (p<0.05). Estimated genomic risk scores paradoxically predicted better prognosis in African American vs European American men for Oncotype DX (p<0.001), with no significant difference for Prolaris or Decipher.

### Androgen deprivation therapy and resistance mechanisms

Multiple studies studied androgen deprivation therapy (ADT) and mechanisms of resistance. (30) analyzed 18 polymorphisms in 12 androgen and estrogen pathway genes in 645 advanced prostate cancer patients treated with ADT. Survival tree analysis identified higher-order SNP-SNP interactions between AKR1C3 rs12529 and AR-CAG repeat length (HR = 9.11 for high-risk node, p=0.001). (17) established an androgen-independent LNCaP-AI cell line and demonstrated that Wnt pathway activation drives androgen-independent growth. LEF1 and WNT5A were identified as direct targets of AR by chromatin immunoprecipitation. (25) investigated TGFBR2-875G>A in 1,765 subjects. TGFBR2-875GG homozygous patients had lower TGFBR2 mRNA expression (AA/AG: 2^-ΔΔCT=1.5, p=0.016) and were associated with shorter time to castration resistance after ADT (86.7 vs 102.4 months, log-rank p=0.032; multivariate HR = 1.47, p=0.024). (40) reviewed the role of testosterone in treating castration-resistant prostate cancer, specifically Bipolar Androgen Therapy (BAT). The ongoing Phase 2 TRANSFORMER trial is comparing BAT to enzalutamide for men with low-volume metastatic CRPC.

### Radiotherapy and PSMA-targeted therapy

(31) investigated copy number alterations in DNA damage response sensor genes as predictive biomarkers for radiotherapy outcome. NBN gains were the most frequent genomic alteration (14.4% in IGRT cohort, 11% in surgery cohort) and were associated with overall genomic instability (p<0.0001). (29) identified miR-106a as a novel regulator of radioresistance through targeting LITAF and upregulating ATM. miR-106a overexpression increased clonogenic survival (p<0.05) and reduced senescence (p<0.001). (41) comprehensively reviewed PSMA-targeted therapy for castration-resistant prostate cancer. PSMA is a transmembrane glycoprotein that exhibits significantly high expression in most prostate cancers, particularly at advanced stages (100-1000x higher than normal prostate). ¹^77^Lu-PSMA-617 demonstrated a significant improvement in median overall survival (15.3 vs 11.3 months, p<0.001) in the phase III VISION trial, becoming the first FDA-approved radiopharmaceutical for mCRPC. ^68^Ga-PSMA PET/CT achieves a 97.5% detection rate for primary lesions and identifies 56% of small metastatic foci (<5 mm) missed by conventional imaging. The review also covered PSMA-targeted immunotherapies (bispecific T-cell engagers, CAR-T therapy), antibody-drug conjugates, and small molecule inhibitors.

### Bone health management, exercise and nutritional interventions

(42) provided a comprehensive review of therapeutic agents for protection bone health in prostate cancer patients. Denosumab (60mg subcutaneously every 6 months) significantly increased bone mineral density and reduced new vertebral fractures (1.5% vs 3.9%, RR = 0.38, p=0.006) in men receiving ADT for non-metastatic hormone-sensitive prostate cancer. For CRPC patients with bone metastases, denosumab 120mg every 4 weeks was superior to zoledronic acid for time to first skeletal-related event (20.7 vs 17.1 months, HR = 0.82, p=0.008). Zoledronic acid reduced skeletal-related events compared with placebo (38% vs 49%, p=0.028). Radium-223 demonstrated improved overall survival (14.9 vs 11.3 months, HR = 0.70, p<0.001) and delayed time to first symptomatic skeletal event (15.6 vs 9.8 months, HR = 0.66, p<0.001). (38) presented a pilot randomized controlled trial protocol investigating periodized resistance training combined with protein supplementation in prostate cancer survivors receiving ADT. The intervention employed a 2×2 factorial design with 32 participants allocated to resistance training plus protein supplementation, resistance training alone, protein supplementation alone, or control. The periodization model progressed from 60-67% 1RM (weeks 1-4) to 70-83% 1RM (weeks 5-12). This represents an innovative supportive care approach to countering the deleterious side effects of ADT, including sarcopenic obesity and metabolic syndrome.

### Risk stratification and personalized management

Multiple studies developed tools for risk stratification to guide personalized prostate cancer management. (23) developed a genetic risk score (GRS) from 16 SNPs in 912 Korean patients (**Table 2**). The GRS was significantly higher in men experiencing biochemical recurrence compared with non-BCR patients (-1.21 vs -2.43, p<0.001). (33) developed an ethnic-specific PRS for clinically significant prostate cancer in Korean males. The best-performing model comprised 29 SNPs with an AUC of 0.700 (95% CI: 0.667-0.734). (18) developed and validated a genomic classifier (gSVI) to predict seminal vesicle invasion using 15,889 patients. In the biopsy validation cohort, gSVI combined with the MSKCC nomogram had superior AUC (0.86) compared with either nomogram individually (0.81). (34) characterized the relationship between tumor volume and genomic risk in 38,921 men. In grade group 1 disease, there was no correlation between percent positive cores and genomic risk (r=0.01, 95% CI: -0.001 to 0.03). In contrast, gradually positive correlations were observed in grade groups 2 (r=0.07), 3 (r=0.15), 4 (r=0.20), and 5 (r=0.25). Multivariable analysis confirmed no association between genomic risk and percent positive cores for grade group 1 (p=0.177), supportive to active surveillance for most grade group 1 patients regardless of tumor volume.

**Table 2:** Effect size and grade.

No	Author	Year	Study design	Intervention	Comparator	Effect size	Risk of bias	GRADE
1	(10)	2025	Multi-cohort GWAS with Prospective Cohort Validation	Radiotherapy for non-metastatic prostate cancer	PRSlow (remaining 90%)	Binarized PRS (HR for PRShigh): Hematuria: HR = 2.12 (p=0.002, adjusted); Retention: HR = 1.85 (p=0.009); Frequency: HR = 1.45 (p=0.07); UKB: OR = 2.15 (p=0.026)	Low to Moderate	Moderate
2	(11)	2020	*In vitro* Laboratory Study (Cell-based Experimental Study)	DTX3L overexpression (pcDNA-DTX3L transfection)	Vector control (pcDNA 4.1); shRNA negative control	DTX3L overexpression: Increased survival (p<0.01); DTX3L knockdown: Decreased survival (p<0.01); DTX3L overexpression: ↓γH2AX, ↑Rad51 (p<0.01); DTX3L knockdown: ↑γH2AX	High (*in vitro* study only, no *in vivo* validation, no clinical data, small sample size, potential for selective reporting)	Very Low (*in vitro* study; no clinical translation; cannot be used for clinical recommendations)
3	(12)	2017	Retrospective Case-Control Study with Prospective Follow-up	N/A (Observational genetic association study)	N/A (Case-control design)	Key significant associations (p<0.05): rs743572 (CYP17A1): PSA>10 ng/mL OR = 3.017, p=0.032; D’Amico risk OR = 3.856, p=0.016; rs6162 (CYP17A1): PSA>10 OR = 3.129	Moderate (retrospective design, limited sample size, multiple testing concerns, heterogeneity in treatments)	Low to Moderate (hypothesis-generating; requires validation in larger cohorts)
4	(13)	2020	Retrospective Cohort (Targeted Sequencing Study)	N/A (Observational genomic study)	N/A (No comparator group)	Key significant findings: PGA (highest quartile): HR = 13.45, p=0.002; TP53 mutations: HR = 9.45, p=0.002; CDKN1B deep deletions: HR = 6.66, p=0.026	Moderate (retrospective, no grade-matched EA comparator, limited validation, overfitted models)	Low to Moderate (novel findings; requires validation in independent AA cohorts)
5	(14)	2022	*In vitro* and *In vivo* Experimental Study	Proton beam irradiation (single dose: 4–8 Gy; fractionated: 24 Gy in 5 fractions)	Photon irradiation (single dose: 4–8 Gy; fractionated: 26 Gy in 5 fractions)	Key findings: Reduced sphere formation with protons (p<0.05); Lower RBE in ALDH+ vs ALDH- cells (p<0.05); Reduced plasticity rates with protons (p=0.03)	Moderate (*in vitro* focused; limited *in vivo* data; high variability; limited proton beam availability)	Low to Moderate (preclinical; hypothesis-generating; requires clinical validation)
6	(15)	2020	Retrospective Cohort (Biomarker Study)	N/A (Observational biomarker study)	N/A (No comparator group)	Key significant findings: GOLM1 overexpression: Extracapsular extension FC = 14.3 (p=0.031); Seminal vesicle invasion FC = 23.2 (p=0.004); pT3+ FC = 11.0 (p=0.041)	Moderate (retrospective design; limited sample size; no validation cohort)	Low to Moderate (promising biomarkers; requires validation in larger prospective cohorts)
7	(16)	2020	Cross-Sectional Genomic Study (Retrospective)	N/A (Observational genomic comparison study)	N/A (Race-based comparison)	Key significant findings: 48% (29/60) genes differentially expressed by race (p<0.05); Oncotype DX: EAM higher risk than AAM (p<0.001); Prolaris: No significant difference (p>0.05)	Moderate (no long-term follow-up; Nanostring platform vs commercial assays; approximated risk scores)	Low to Moderate (race-based differences identified; clinical impact requires validation with outcome data)
8	(17)	2020	*In vitro* Laboratory Study + Bioinformatics Analysis	AR signaling modulation (DHT stimulation, CDX/AR antagonist treatment); Wnt pathway inhibition (KY02111, XAV-939)	Androgen-dependent LNCaP cells	Key findings: AR transcriptional activity reduced in LNCaP-AI (p<0.001); 1,282 upregulated and 854 downregulated genes during ADT; LEF1 and WNT5A direct AR targets (ChIP confirmed)	Moderate (*in vitro* only; limited clinical validation; bioinformatics with public data)	Low (preclinical; requires *in vivo* and clinical validation)
9	(18)	2020	Retrospective Cohort (Biomarker Development and Validation)	N/A (Observational genomic study)	N/A (No comparator group)	Key findings: gSVI + MSKCC nomogram AUC = 0.86 (vs MSKCC alone 0.81, Partin alone 0.81); gSVI OR = 2.74-2.77 per 0.1 unit increase (p<0.001); 581-gene signature	Moderate (retrospective; no prospective validation; clinical utility not yet demonstrated)	Moderate (validated in large cohorts; but requires prospective validation)
10	(19)	2023	*In vitro* Laboratory Study + Human Tissue Analysis	Androgen treatment (R1881); siRNA knockdown (sortilin, AS160); Glucose modulation; Lipid treatment	Vehicle control; Scrambled siRNA; Normal glucose (5.5 mM)	Key findings: Sortilin high in low-grade PCa; syndecan-1 high in high-grade (p<0.05); Sortilin knockdown ↓ glucose uptake (p<0.001); R1881 ↑ sortilin, GLUT1, AS160 (p<0.05)	Moderate (*in vitro* focused; limited clinical validation; cell line limitations)	Low to Moderate (preclinical; novel mechanism; requires *in vivo* validation)
11	(20)	2022	Retrospective Cohort + *In vitro* + Liquid Biopsy	RAI2 knockdown (shRNA); AR knockdown; AR inhibition (bicalutamide, enzalutamide, hormone deprivation)	Non-target shRNA; vehicle control	Key findings: High RAI2 protein correlated with early BCR (p=0.0024); RAI2 correlated with AR, K8/18/19, E-cadherin (p<0.001-0.007); RAI2 mRNA in CTCs correlated with AR, AR-V7, PSA	Moderate (retrospective; limited CTC sample size; semiquantitative PCR)	Low to Moderate (biomarker potential; requires prospective validation)
12	(21)	2023	Retrospective Cohort + *In vitro* + *In vivo*	FSCN1 knockdown (siRNA/shRNA); AR overexpression; DHT treatment; Bicalutamide treatment	Control siRNA/shRNA; Vehicle control	Key findings: FSCN1 high in AR-negative cells and NEPC tissues; FSCN1 inversely correlated with KLK3 (r=-0.21 to -0.57, p<0.05); DHT ↓ FSCN1 in VCaP (p<0.0001)	Moderate (retrospective; small NEPC cohort; cell line models)	Low to Moderate (novel biomarker and therapeutic target; requires clinical validation)
13	(22)	2021	*In vitro* + *In vivo* Laboratory Study	Castration-resistant cell line development; AR antagonist treatment (enzalutamide, bicalutamide); DHT treatment	Parental androgen-dependent lines (LAPC4, VCaP, LNCaP)	Key findings: LAPC4-CR and VCaP-CR show CR growth *in vitro*/vivo; Resistant to enzalutamide and bicalutamide; VCaP-CR sensitive to high-dose DHT (growth arrest)	Low to Moderate (preclinical; cell line models; no clinical validation)	Low (preclinical; resource for CRPC research)
14	(23)	2017	Prospective Cohort (Genetic Association Study)	N/A (Observational genetic study)	N/A (No comparator group)	Key findings: 16 SNPs associated with BCR (p<0.001); GRS higher in BCR vs non-BCR (-1.21 vs -2.43, p<0.001); 10-year BCR-free survival: 46.3% (high GRS) vs 81.8% (low GRS)	Moderate (prospective but single institution; no validation cohort; multiple testing concerns)	Low to Moderate (potential biomarker; requires validation in independent cohorts)
15	(24)	2019	*In vitro* + *In vivo* Laboratory Study + Human Tissue Analysis	HMGB1 knockdown (shRNA/siRNA); HMGB1 overexpression (lentiviral); BRG1 modulation	sh-Ctrl; vector control	Key findings: HMGB1 knockdown ↓ proliferation, migration, invasion (p<0.05); HMGB1 overexpression ↑ aggressive behavior (p<0.05); HMGB1 interacts with BRG1	Moderate (preclinical; limited clinical validation; small tissue cohort)	Low to Moderate (mechanistic; potential therapeutic target; requires clinical validation)
16	(25)	2013	Retrospective Case-Control + Prospective Follow-up Cohort	TGFBR2-875G>A genotyping; ADT treatment	N/A (Genetic association study)	Key findings: TGFBR2-875GG associated with Gleason ≥8 (OR = 1.51, p=0.019); TGFBR2-875GG associated with shorter time to CR (86.7 vs 102.4 months, p=0.032)	Moderate (retrospective design; limited sample for gene expression)	Low to Moderate (CRPC predictive model; requires validation)
17	(26)	2018	Retrospective Cohort (Tissue Microarray Study)	N/A (Observational biomarker study)	BPH and normal prostate tissues	Key findings: Paxillin higher in PCa vs BPH/normal (p<0.001); Paxillin associated with PSA, Gleason, T stage, LNM, PSM, ECE, SVI (p<0.05)	Moderate (retrospective; single center; no validation cohort; no functional studies)	Low to Moderate (prognostic biomarker; requires validation)
18	(J. 27)	2020	Retrospective Cohort (Tissue Microarray Study)	N/A (Observational biomarker study)	BPH tissues	Key findings: LIMK1 higher in PCa vs BPH (77.1% vs 26.0%, p<0.001); LIMK1 higher in positive LN vs paired PCa (p=0.002); LIMK1 associated with prostate volume, PSA, PSAD, Gleason	Moderate (retrospective; single center; no validation cohort)	Low to Moderate (prognostic biomarker; requires validation)
19	(28)	2024	*In vitro* + *In vivo* + PDX Analysis	Cabazitaxel; Ispinesib (kinesin inhibitor); CW069 (HSET inhibitor); SLCO1B3 knockdown	Vehicle control; Untreated cells	Key findings: C4-2BDR resistant to cabazitaxel (p<0.05); Cabazitaxel drives MET (↑E-cadherin, ↑IGFBP-3); HSET-Bcl-2 interaction enriched with cabazitaxel (Co-IP)	Moderate (preclinical; limited *in vivo*; small PDX cohort)	Low (preclinical; requires clinical validation)
20	(29)	2018	*In vitro* + *In vivo* + Bioinformatics	miR-106a overexpression; LITAF siRNA knockdown; KU-55933 (ATM inhibitor)	Control mimic; Control siRNA; DMSO vehicle	Key findings: miR-106a ↑ in PCa vs normal (p=7.66e-18); miR-106a ↑ in high-grade PCa (p=0.05); miR-106a overexpression ↑ clonogenic survival (p<0.05), ↑ proliferation (p<0.05)	Low-Moderate (preclinical; limited clinical validation; TCGA analysis)	Low to Moderate (novel mechanism; requires clinical validation)
21	(30)	2013	Retrospective Cohort (Genetic Association Study)	ADT (orchiectomy or LHRH agonist ± antiandrogen)	N/A (Observational genetic study)	Key findings: AKR1C3 rs12529 associated with PCSM (CC vs GG/GC: HR = 5.23, p=0.006); AR-CAG repeat length associated with PCSM (p-trend=0.041); Combined unfavorable genotypes	Moderate (retrospective; single Asian population; multiple testing)	Low to Moderate (potential biomarkers; requires validation in other populations)
22	(31)	2014	Retrospective Cohort (Biomarker Study)	Image-guided radiotherapy (IGRT) (dose 60-79.8 Gy)	Radical prostatectomy (RadP)	Key findings: NBN gain most frequent DDR alteration (IGRT 15.1%, RadP 11.6%); NBN gain associated with higher PGA (p<0.0001); NBN gain predicted worse 5-yr bRFR after IGRT	Moderate (retrospective; single center; limited cohort sizes)	Low to Moderate (promising predictive biomarker; requires validation)
23	(32)	2014	Prospective Cohort (RNA-seq Study)	ADT (androgen-deprivation therapy)	Pre-ADT tumor biopsies (paired)	Key findings: 774 upregulated, 755 downregulated genes post-ADT (FDR<0.05); Wnt signaling pathway upregulated (29 genes, >2-fold enrichment); b-catenin overexpression in 16/29 (55%)	Moderate (small sample size; no independent validation; limited functional studies)	Low to Moderate (hypothesis-generating; Wnt pathway as therapeutic target)
24	(33)	2022	Retrospective Cohort (Genetic Association Study)	N/A (Observational genetic study)	N/A (No comparator group)	Key findings: rs72725879 (PRNCR1) most significant: OR = 0.597, p=4.3E-45; 31 SNPs selected for PRS; Best model: 29 SNPs, AUC = 0.700 (95% CI: 0.667-0.734), Sensitivity=0.672	Moderate (retrospective; Asian population only; limited external validation)	Low to Moderate (promising PRS; requires multi-ethnic validation)
25	(34)	2023	Retrospective Cohort (Registry Study)	N/A (Observational genomic study)	N/A (No comparator group)	Key findings: GG1: PPC-GC correlation r=0.01 (95% CI: -0.001 to 0.03), p=NS; GG2: r=0.07 (0.05-0.08); GG3: r=0.15 (0.12-0.17); GG4: r=0.20 (0.15-0.24); GG5: r=0.25 (0.19-0.31); Multivariable	Moderate (retrospective; no long-term outcomes; limited race data)	Low to Moderate (supports AS for GG1 regardless of volume)
26	(35)	2014	Retrospective Cohort (Genetic Association Study)	N/A (Observational genetic study)	N/A (No comparator group)	Key findings: rs16901979 (8q24): Carriers had larger tumor volume (5.1 vs 3.6 cc, p=0.002; multivariate coefficient 1.6, p=0.01); rs6983267 (8q24): Carriers had larger tumor volume	Moderate (retrospective; white only; limited power)	Low to Moderate (SNPs as bona fide markers; detection bias identified)
27	(36)	2022	Retrospective Cohort + *In vitro* + Bioinformatics	N/A (Observational genetic study; TNFRSF13B knockdown *in vitro*)	N/A (No comparator group)	Key findings: TNFRSF13B rs4792800 GG vs AA/AG: HR = 1.78 (95% CI: 1.16-2.71, p=0.008, q=0.019); G allele associated with higher TNFRSF13B expression (p=0.038)	Moderate (retrospective; single Asian population; limited sample size)	Low to Moderate (TNFRSF13B as novel biomarker; requires validation)
28	(37)	2017	*In vitro* + Bioinformatics	TPX2 siRNA knockdown	si-CTL (scrambled siRNA)	Key findings: TPX2 ↑ in PCa vs normal (p<0.001); TPX2 correlated with Gleason score (R = 0.467, p<0.001); TPX2 silencing ↓ proliferation, colony formation, spheroid formation (p<0.05)	Moderate (*in vitro* only; small tissue cohort; no *in vivo* validation)	Low to Moderate (preclinical; TPX2 as therapeutic target; requires validation)
29	(38)	2017	Pilot Randomized Controlled Trial (2×2 factorial design)	RTPS: Periodized resistance training (3x/week, 45 min sessions) + 50g/day whey protein supplementation	PS: Protein supplementation only (50g/day whey protein)	Not yet reported (study ongoing at time of publication)	Unclear (pilot study, no blinding, allocation concealment not specified)	Low (pilot study, protocol paper only

### Biomarker-based management

(15) evaluated cell-free mRNA markers in plasma from 60 patients. Three genes (GOLM1, NKX3-1, TRPM8) were associated with high-risk disease (Gleason ≥8 and/or tumor stage ≥pT3a). The 3-gene panel identified high-risk cases with 85% sensitivity and 58% specificity, with an AUC of 0.76, outperforming biopsy Gleason score (AUC = 0.66) and PSA >20 ng/mL (AUC = 0.66). Addition of the mRNA panel to clinical factors increased sensitivity from 15% to 88%. Zheng et al. (26) evaluated Paxillin expression in 386 PCa specimens. Paxillin was an independent risk factor for lymph node metastasis (OR = 2.108, p<0.001) and biochemical recurrence (HR = 3.322, p<0.001). AUC for lymph node metastasis prediction was 0.723 for Paxillin vs 0.692 for Gleason score, demonstrating improved risk stratification. (M. 39) evaluated LIMK1 expression in 279 PCa specimens. LIMK1 was an independent risk factor for lymph node metastasis (OR = 2.289, p<0.001) and biochemical recurrence (HR = 2.933, p<0.001). LIMK1 high expression was associated with shortened biochemical-free survival (p<0.001). (41) highlighted quality of life improvements with PSMA-targeted therapy. In the ENZA-p trial, patients receiving ¹^77^Lu-PSMA-617 plus enzalutamide demonstrated significantly prolonged preservation of physical function (mTTD: 10.64 vs 3.42 months, HR = 0.51, p<0.0001) and global health/QoL (mTTD: 8.71 vs 3.32 months, HR = 0.47, p=0.0001). (42) noted that skeletal-related events are associated with substantial pain and reduced quality of life, emphasizing the importance of bone health management for patient well-being. Denosumab and bisphosphonates can ameliorate bone pain in addition to preventing skeletal events.

The etiology of prostate cancer is multifactorial, involving a complex interplay of genetic, molecular, hormonal, and environmental factors. Evidence from the included studies reveals several key etiological mechanisms driving prostate cancer initiation, progression, and therapeutic resistance. Several studies identified specific genetic variants associated with prostate cancer risk and aggressiveness. 25 investigated the TGFBR2-875G>A (rs3087465) polymorphism in a Portuguese cohort of 891 prostate cancer patients and 874 healthy controls. The polymorphism did not increase overall prostate cancer susceptibility (OR = 1.05, 95% CI 0.87–1.28; P = 0.645); however, GG homozygotes demonstrated significantly lower TGFBR2 mRNA expression (1.5-fold lower than AA/AG carriers, P = 0.016) and were associated with higher Gleason scores (OR = 1.51, 95% CI 1.07–2.16; P = 0.019). This suggests that while the polymorphism may not confer initial susceptibility, it significantly influences tumor aggressiveness through altered gene expression. The androgen receptor (AR) pathway remains central to prostate cancer etiology. (30) demonstrated that genetic variations within the androgen/estrogen pathway genes, particularly AKR1C3 rs12529 and AR CAG repeat length, were independent predictors of prostate cancer-specific mortality (PCSM) following androgen deprivation therapy (ADT). Patients carrying two unfavorable genotypes had substantially poorer survival (adjusted HR = 13.7, 95% CI 3.60–52.4; P < 0.001), highlighting the critical role of inherited genetic variation in AR pathway signaling in determining disease outcomes.

(24) identified High Mobility Group Box 1 (HMGB1) as a serious etiological factor in prostate cancer progression. HMGB1 overexpression significantly promoted cell proliferation, invasion, migration, epithelial-mesenchymal transition (EMT), and Akt phosphorylation in prostate cancer cell lines (PC-3, LNCaP, 22Rv1). HMGB1 expression positively correlated with BRG1 expression (r = 0.574, P < 0.001) and was associated with higher Gleason scores and advanced clinical stage in 64 prostate cancer tissues. Mouse xenograft models demonstrated that HMGB1 knockdown significantly prolonged survival (log-rank P = 0.026), confirming its role as a driver of tumor aggressiveness and metastasis. (26) established that Paxillin expression was significantly higher in prostate cancer tissues compared to normal prostate and benign prostatic hyperplasia (BPH) tissues, with expression increasing progressively with higher Gleason Grade Groups. Paxillin expression was significantly elevated in patients with lymph node metastases and in metastatic lymph nodes compared to matched primary tumors (multivariable Cox analysis: HR = 3.322, 95% CI 1.987–5.551, P < 0.001). Equally, (M. 39) identified LIMK1 as a key etiological factor, with expression significantly higher in prostate cancer than BPH tissues (77.1% vs 26.0%; P < 0.001) and significantly elevated in metastatic lymph nodes compared to matched primary tumors (P = 0.002). High LIMK1 expression independently predicted lymph node metastasis (OR = 2.289, 95% CI 1.694–3.092, P < 0.001), suggesting its critical role in metastatic progression.

(21) clarified the role of FSCN1 (Fascin-1) in neuroendocrine differentiation, a hallmark of aggressive prostate cancer. FSCN1 was markedly upregulated during neuroendocrine differentiation and predominantly expressed in neuroendocrine prostate cancer tissues. The activated androgen receptor directly suppressed FSCN1 transcription by binding to its regulatory region, while AR antagonists relieved this repression. Silencing FSCN1 reduced neuroendocrine characteristics and impaired cell migration, indicating that dysregulation of AR-FSCN1 signaling contributes to neuroendocrine prostate cancer pathogenesis. (20) demonstrated that low RAI2 gene expression was associated with hypoxia and genomic instability in prostate cancer. Low RAI2 expression correlated with increased mutation count (P = 4.412 × 10^-5^), increased tumor mutational burden (P = 9.356 × 10^-4^), and increased microsatellite instability (P = 7.758 × 10^-^³). High RAI2 protein expression significantly correlated with androgen receptor expression and epithelial differentiation markers, while RAI2 depletion increased expression of androgen-responsive genes, supporting RAI2 as a transcriptional corepressor of androgen signaling.

### Risk of bias distribution

The quality assessment of included studies was performed using appropriate tools based on study design: Cochrane RoB 2 for randomized trials, Newcastle-Ottawa Scale for observational studies, and SYRCLE’s Risk of Bias tool for animal/preclinical studies. The GRADE framework was applied to evaluate the overall certainty of evidence for each body of evidence.

Summary of Risk of Bias Assessments: For human observational studies assessed using the Newcastle-Ottawa Scale, the following domain-level patterns were observed: Low Risk: Prospective cohort studies with clear case definitions, representative cohorts, and adequate selection of controls. Moderate Risk: Retrospective cohort studies with potential selection bias due to hospital-based sampling or exclusion criteria.

### GRADE framework for certainty of evidence

The GRADE (Grading of Recommendations Assessment, Development and Evaluation) framework was applied to evaluate the overall certainty of evidence for each body of evidence. GRADE assessments considered study design, risk of bias, inconsistency, indirectness, imprecision, and publication bias.

Overall Evidence was Moderate Certainty Evidence: (30) Prospective cohort with large sample, multivariable adjustment, strong associations (HR up to 13.7). (23) Large prospective cohort with robust adjustment and consistent findings and (10) Multicentered prospective cohorts with external validation and standardized outcomes. These studies provide moderately certain evidence that genetic factors significantly influence prostate cancer outcomes and treatment toxicity.

Low Certainty Evidence: Most observational cohort studies (M. 25, 26, 36, 39). Translational studies with human tissue validation but limited sample sizes (20, 32). These studies provide suggestive evidence for biomarker associations but require further validation.

## Discussion

The synthesis of these 29 articles underscores the complex, multifactorial etiology of prostate cancer and the rapid evolution of its therapeutic options. Genetic predisposition plays a significant role in both disease susceptibility and treatment response with several studies showing the prognostic value of genetic polymorphisms and gene expression signatures. For instance, variants in TGFBR2 (25) and AKR1C3 (30) were associated with higher Gleason scores, earlier relapse after ADT, and worse cancer associated mortality. Additionally, the use of polygenic risk scores (PRS) demonstrated strong predictive potential, not only for disease fierceness but also for expected treatment related toxicities for example late bladder toxicity post-radiotherapy (10, 36). This means that integrating germline genetics into clinical decision making could enhance risk stratification and personalization of therapy.

Although androgen deprivation therapy (ADT) remains a cornerstone of systemic treatment for advanced prostate cancer, its use should be guided by established clinical benefit and individualized risk–benefit considerations. The IMAGINE study, which audited 6,598 men undergoing radical prostatectomy across 187 hospitals in 31 European countries, found 98% adherence to European Association of Urology recommendations against neoadjuvant ADT before prostate cancer surgery (43) However, inappropriate preoperative ADT use persisted, particularly among patients with high-risk and locally advanced disease, reflecting continued variation in treatment practice. This is clinically important given the potential metabolic, cardiovascular, skeletal, psychological, and sexual adverse effects associated with ADT and the absence of established oncological benefit for routine neoadjuvant use before surgery. These findings reinforce the importance of evidence-based and individualized selection of ADT, alongside consideration of factors that influence treatment response and progression to castration-resistant disease.

The review also provided substantial evidence from preclinical models showing critical molecular pathways driving prostate cancer progression and therapy resistance. The Wnt/β-catenin pathway was a key mechanism contributing to castration resistant prostate cancer (CRPC) following ADT (32). The evolution toward precision medicine is clearly reproduced in the articles. The identification of actionable biomarkers such as HMGB1, LIMK1, Paxillin, and RAI2 (20; J. 24, 26, 27) has provided understandings into tumor aggressiveness and prognostic outcomes. Nevertheless, a significant gap remains between these preclinical discoveries and their translation into clinical practice. A major restriction emphasized across studies is the predominance of retrospective, single-center observational designs and the dependence on preclinical models that may not fully replicate human tumor heterogeneousness. The systematic review on bipolar androgen therapy (40) and the findings on the utility of denosumab versus zoledronic acid (42) further emphasize that while treatment patterns are advancing, robust, prospective, and comparative trials are crucial to establish the accurate clinical efficacy of emerging therapies. This is also evident in the recent study by (44) highlights emerging and evolving therapeutic modalities having high chance for their translational abilities over challenges such as resistance.

The identification of germline and somatic genetic alterations provides insight into prostate cancer etiology. (12) demonstrated that variants in sex hormone pathway genes (CYP17A1, LHCGR, ESR2) are connected with aggressive disease characteristics, signifying that genetic variation in androgen and estrogen metabolism backs to prostate cancer risk and progression. The finding that specific genotype combinations (GTGTAACA) confer increased risk for D’Amico classification, higher PSA, and Gleason ≥7 supports a polygenic model of disease etiology. (13) identification of CDKN1B deep deletions (6.3%) and TP53 mutations (3.9%) as predictors of metastasis in African American men addresses an important gap in understanding prostate cancer etiology in high-risk populations. The finding that percent genome alteration in the highest quartile predicts metastasis (HR = 13.45) suggests that overall genomic instability is a powerful driver of aggressive disease. This is particularly significant given the higher incidence and mortality of prostate cancer in African American men, highlighting the need for race-specific etiologic research.

(33) development of an ethnic-specific PRS for clinically significant prostate cancer demonstrates that the cumulative effect of multiple risk alleles contributes to disease etiology. The identification of rs72725879 in PRNCR1 as the most significant SNP (OR = 0.597, p=4.3×10^-45^) and the 4.92-fold increased risk in the top 5th percentile of PRS provides evidence for polygenic inheritance in prostate cancer. (16) finding that 48% of genes in commercial prognostic panels are differentially expressed by race highlights the importance of considering race and ethnicity in prostate cancer etiology. The paradoxical finding that Oncotype DX estimated better prognosis in African American vs European American men (p<0.001) is concerning and suggests that race-specific calibration of genomic tools is needed. This supports the hypothesis that prostate cancer has distinct molecular subtypes that vary by race, contributing to disparate outcomes.

The studies reviewed provide a comprehensive understanding of ADT and mechanisms of resistance. (30) finding that AKR1C3 rs12529 (HR = 5.23) and AR-CAG repeat length (p-trend=0.041) predict prostate cancer-specific mortality after ADT supports the concept that genetic variation in androgen metabolism influences treatment response. The 13.7-fold increased risk in individuals carrying two unfavorable genotypes suggests that genetic testing could identify patients at highest risk of poor outcomes and guide treatment intensification. (32) RNA-seq study demonstrated that Wnt/β-catenin pathway activation (29 genes, >2-fold enrichment) occurs after ADT in clinical samples. The confirmation of β-catenin overexpression in 55% of CRPC pairs and correlation with AR expression (r=0.440, p=0.017) suggests that Wnt pathway activation is a clinically relevant resistance mechanism. Luo et al.’s finding that Wnt pathway inhibitors specifically target androgen-independent cell growth (p<0.001) provides a potential therapeutic strategy for overcoming ADT resistance. (25) finding that TGFBR2-875GG predicts shorter time to castration resistance (86.7 vs 102.4 months, HR = 1.47) and that addition of genetic information improves predictive models (c-index 0.683 to 0.759) supports the integration of genetic biomarkers into ADT management.

(40) review of Bipolar Androgen Therapy (BAT) presents a novel therapeutic approach that exploits the paradoxical growth-suppressive effects of supraphysiologic testosterone in CRPC. The mechanistic rationale disruption of AR recycling and induction of DNA damage via topoisomerase IIβ recruitment is supported by preclinical evidence. The TRANSFORMER pilot study’s finding of 50% PSA decline and 100% response to subsequent AR-targeting therapies suggests BAT may re-sensitize tumors to antiandrogens, offering a promising new therapeutic strategy. (41) comprehensive review of PSMA-targeted therapy demonstrates the transformative potential of theranostics in prostate cancer treatment. The approval of ¹^77^Lu-PSMA-617 based on the VISION trial (mOS 15.3 vs 11.3 months, p<0.001) represents a major advance in mCRPC treatment. The ability to use PSMA PET/CT for patient selection (97.5% detection rate for primary lesions) enables a “see-and-treat” paradigm that personalizes therapy. The review also highlights emerging PSMA-targeted modalities including bispecific T-cell engagers, antibody-drug conjugates, and CAR-T therapy, suggesting that the therapeutic landscape will continue to evolve.

(31) finding that NBN gain predicts radiotherapy failure (HR = 3.28) but not surgery failure provides strong evidence for treatment personalization based on genomic biomarkers. The *in vitro* correlation between NBN expression and radioresistance (r²=0.871) supports the biological rationale. This is the first study to demonstrate a predictive rather than merely prognostic biomarker for radiotherapy in prostate cancer, with potential to guide treatment selection between surgery and radiotherapy. (29) identification of miR-106a as a regulator of radioresistance through the LITAF→ATM axis provides a molecular mechanism that could be therapeutically targeted. The finding that ATM inhibition with KU-55933 re-sensitizes cells to radiation suggests a potential combination strategy to overcome radioresistance. The development of multiple risk stratification tools supports personalized prostate cancer management. Oh et al.’s GRS (AUC improvement from 0.844 to 0.880, p=0.0026), Song et al.’s ethnic-specific PRS (AUC = 0.700, 4.92-fold risk in top 5%), and Hall et al.’s gSVI (AUC improvement from 0.81 to 0.86) demonstrate that genomic information adds value beyond clinical factors. (34) finding that tumor volume is not associated with genomic risk in grade group 1 disease (r=0.01, p=NS) is clinically significant as it supports active surveillance for most patients with low-grade disease regardless of volume. This addresses the controversy regarding tumor volume in low-risk patients and provides evidence-based support for conservative management.

(15) 3-gene circulating mRNA panel (GOLM1, NKX3-1, TRPM8) demonstrates the potential of liquid biopsy for risk stratification (AUC = 0.76 vs 0.66 for PSA/Gleason). The identification of these genes as predictors of high-risk disease provides a non-invasive approach for identifying patients needing more aggressive management. (26) finding that Paxillin predicts lymph node metastasis (AUC = 0.723) and biochemical recurrence (HR = 3.322) and Huang et al.’s finding that LIMK1 predicts lymph node metastasis (OR = 2.289) and biochemical recurrence (HR = 2.933) add to the growing panel of tissue-based biomarkers for risk stratification. (41) finding that PSMA-targeted therapy improves quality of life (mTTD physical function: 10.64 vs 3.42 months, HR = 0.51, p<0.0001) and Miller et al.’s emphasis on bone health management highlight the importance of quality of life as a management goal. The evidence that denosumab and bisphosphonates can ameliorate bone pain while preventing skeletal events supports their use not only for skeletal protection but also for symptom management.

Moreover, the current suggests many molecular targets are rated as Low according to the GRADE framework. This is principally due to methodological restrictions, including lack of external validation, small sample sizes, and indirectness of evidence from preclinical studies. Despite these restrictions, there is consistent evidence supportive the role of genetic and molecular biomarkers in improving prognostic precision over traditional clinicopathological variables alone. For instance, adding the TGFBR2 genotype enhanced the predictive model’s c-index from 0.683 to 0.759 (25), and the addition of a GRS significantly improved the AUC for predicting BCR (23). This proposes that a multi parametric approach merging clinical factors with genetic and molecular data embraces the most potential for advancing precision oncology in prostate cancer.

### Strengths and limitations

This systematic review has several strengths, including the inclusion of 31 primary studies with diverse designs, comprehensive coverage of prostate cancer etiology, risk factors, treatment, therapy, and management, and alignment with review keywords. The inclusion of studies from multiple geographic regions (USA, Europe, Asia) provides a global perspective.

Limitations include the heterogeneity of study designs, precluding meta-analysis. Most included studies were retrospective, with only four prospective cohort studies. The small sample sizes of some studies [(38) protocol with 32 planned participants, Rajan’s RNA-seq with 7 patients] limit generalizability. The predominance of studies in Caucasian and Asian populations limits applicability to African American and other populations. The inclusion of narrative reviews (40–42) as secondary sources introduces potential citation bias.

## Conclusion

This systematic review of 29 primary studies presents detailed evidence on the etiology, risk factors, treatment, therapy, and management of prostate cancer in developed and developing countries with recommendations for future research. Prostate cancer etiology is multifaced and slinked to genetic predisposition, race/ethnicity, age, and lifestyle factors. Modifiable risk factors such as diet, obesity, and smoking provide opportunities for prevention. Androgen blocking therapy is still the common advanced prostate cancer treatment. Risk stratification using polygenic risk scores, genetic risk scores, genomic classifiers, and tissue-based biomarkers enables personalized management. Active search remains significant for most grade group 1 patients regardless of tumor size, as tumor size is not associated with genomic risk in low-grade disease. It is therefore, important for future research to focus on validating genomic biomarkers in diverse populations, developing race-specific risk models, identifying optimal combination strategies for novel therapies. Investigation of mechanisms of treatment resistance and strategies to overcome them remains a priority for improving outcomes in advanced prostate cancer. Future work in this area should organize large, prospective, multicentered studies that incorporate comprehensive genomic and clinical data to validate promising biomarkers. Also, well designed randomized controlled trials are urgently needed to evaluate the clinical benefit of novel combination therapies and to determine their role in the standard of care. Applying these mechanistic perceptions into clinical practice will need rigorous validation and an ongoing commitment to precision oncology. This will be certifying that the developing therapeutic options are optimally matched to individual patient risk profiles and tumor biology.

## Data Availability

The original contributions presented in the study are included in the article/supplementary material, further inquiries can be directed to the corresponding author/s.

## Glossary

ACM: All-Cause Mortality
ADT: Androgen Deprivation Therapy
AKT: Protein Kinase B (PKB)
ALP: Alkaline Phosphatase
AR: Androgen Receptor
AUC: Area Under the Curve
BAT: Bipolar Androgen Therapy
BCR: Biochemical Recurrence
BFS: Biochemical Recurrence-Free Survival
BPH: Benign Prostatic Hyperplasia
BRCA2: Breast Cancer Gene 2
BRG1: Brahma-Related Gene 1
BSA: Bovine Serum Albumin
CASP8: Caspase-8
CD: Control Diet
ChIP: Chromatin Immunoprecipitation
CHGA: Chromogranin A
CI: Confidence Interval
CRISPR: Clustered Regularly Interspaced Short Palindromic Repeats
CRPC: Castration-Resistant Prostate Cancer
CSS: Cancer-Specific Survival
CTC: Circulating Tumor Cell
DHT: Dihydrotestosterone
DKO: Double Knockout
DNA: Deoxyribonucleic Acid
DTX3L: Deltex E3 Ubiquitin Ligase 3L
EMT: Epithelial-Mesenchymal Transition
ENO2: Enolase 2
ERK: Extracellular Signal-Regulated Kinase
FDR: False Discovery Rate
FFA: Free Fatty Acid
FOLH1: Folate Hydrolase 1
FOXO: Forkhead Box O
FSCN1: Fascin-1
GATA3: GATA Binding Protein 3
GEO: Gene Expression Omnibus
GFRAL: GDNF Family Receptor Alpha-Like
GG: Gleason Grade
GPX2: Glutathione Peroxidase 2
GRADE: Grading of Recommendations Assessment, Development and Evaluation
GRS: Genetic Risk Score
GWAS: Genome-Wide Association Study
Gy: Gray (unit of radiation)
HFD: High-Fat Diet
HMGB1: High Mobility Group Box 1
HR: Hazard Ratio
HPE: Human Prostate Epithelial
HSET: Kinesin-14 (KIFC1)
ID1: Inhibitor of DNA Binding 1
IGF1: Insulin-Like Growth Factor 1
IGFBP3: Insulin-Like Growth Factor-Binding Protein 3
IL: Interleukin
ISUP: International Society of Urological Pathology
KEGG: Kyoto Encyclopedia of Genes and Genomes
Ki-67: Marker of Proliferation Ki-67
KLK3: Kallikrein-Related Peptidase 3 (PSA)
LA: Linoleic Acid
LDH: Lactate Dehydrogenase
LDLR: Low-Density Lipoprotein Receptor
LIMK1: LIM Domain Kinase 1
LITAF: Lipopolysaccharide-Induced TNF Factor
LNCaP: Lymph Node Carcinoma of the Prostate
LNCaP-AI: LNCaP Androgen-Independent
LPL: Lipoprotein Lipase
MD: Mean Difference
MeSH: Medical Subject Headings
MET: Mesenchymal-Epithelial Transition
MIC-1: Macrophage Inhibitory Cytokine-1
miR: MicroRNA
MME: Membrane Metalloendopeptidase
mRNA: Messenger Ribonucleic Acid
MTT: 3-(4,5-Dimethylthiazol-2-yl)-2,5-Diphenyltetrazolium Bromide
NEPC: Neuroendocrine Prostate Cancer
NSG: NOD SCID Gamma (mouse strain)
OA: Oleic Acid
OR: Odds Ratio
OS: Overall Survival
PA: Palmitic Acid
PARP: Poly (ADP-Ribose) Polymerase
PCa: Prostate Cancer
PCSM: Prostate Cancer-Specific Mortality
PDX: Patient-Derived Xenograft
PMS1: PMS1 Homolog 1
PRISMA: Preferred Reporting Items for Systematic Reviews and Meta-Analyses
PRS: Polygenic Risk Score
PSA: Prostate-Specific Antigen
PSMA: Prostate-Specific Membrane Antigen
QC: Quality Control
qRT-PCR: Quantitative Reverse Transcription Polymerase Chain Reaction
RAI2: Retinoic Acid-Induced 2
RCT: Randomized Controlled Trial
RNA: Ribonucleic Acid
RNA-seq: RNA Sequencing
ROBINS-I: Risk Of Bias In Non-randomized Studies - of Interventions
RR: Relative Risk
SD: Standard Deviation
shRNA: Short Hairpin Ribonucleic Acid
siRNA: Small Interfering Ribonucleic Acid
SLCO1B3: Solute Carrier Organic Anion Transporter Family Member 1B3
SMAD7: SMAD Family Member 7
SNP: Single Nucleotide Polymorphism
SRE: Skeletal-Related Event
SYP: Synaptophysin
SYRCLE: Systematic Review Centre for Laboratory Animal Experimentation
TCGA: The Cancer Genome Atlas
TGFBR2: Transforming Growth Factor Beta Receptor 2
TNFRSF13B: Tumor Necrosis Factor Receptor Superfamily Member 13B
URWCI: Urethra-Rectum-Wall-Cancer-Intervention
Wnt: Wingless-Related Integration Site
XAV939: Wnt/β-Catenin Inhibitor
XRCC4: X-Ray Repair Cross-Complementing 4
αSMA: Alpha-Smooth Muscle Actin
β-Catenin: Beta-Catenin
γH2AX: Gamma-H2AX Histone.
